# Engagement of older adults with multimorbidity as patient research partners: Lessons from a patient-oriented research program

**DOI:** 10.1177/2633556521999508

**Published:** 2021-03-17

**Authors:** Maureen Markle-Reid, Rebecca Ganann, Jenny Ploeg, Gail Heald-Taylor, Laurie Kennedy, C McAiney, Ruta Valaitis

**Affiliations:** 1 School of Nursing, McMaster University, Hamilton, Ontario, Canada; 2 Health Research Methods, Evidence and Impact, 62703 Faculty of Health Sciences, McMaster University, Hamilton, Ontario, Canada; 3 Aging, Community and Health Research Unit, 62703 Faculty of Health Sciences, McMaster University, Hamilton, Ontario, Canada; 4 McMaster Institute for Research on Aging, McMaster University, Hamilton, Ontario, Canada; 5 Department of Health, Aging and Society, McMaster University, Hamilton, Ontario, Canada; 6 School of Public Health and Health Systems, 8430 University of Waterloo, Waterloo, Ontario, Canada; 7 Schlegel-UW Research Institute for Aging, 8430 University of Waterloo, Waterloo, Ontario, Canada; 8 Murray Alzheimer Research & Education Program (MAREP), 8430 University of Waterloo, Waterloo, Ontario, Canada

**Keywords:** Patient engagement, patient-oriented research, older adults, multimorbidity, health intervention

## Abstract

**Background:**

Patient “engagement” in health research broadly refers to including people with lived experience in the research process. Although previous reviews have systematically summarized approaches to engaging older adults and their caregivers in health research, there is currently little guidance on how to meaningfully engage older adults with multimorbidity as research partners.

**Objectives:**

This paper describes the lessons learned from a patient-oriented research program, the Aging, Community and Health Research Unit (ACHRU), on how to engage older adults with multimorbidity as research partners. Over the past 7-years, over 40 older adults from across Canada have been involved in 17 ACHRU projects as patient research partners.

**Methods:**

We developed this list of lessons learned through iterative consensus building with ACHRU researchers and patient partners. We then met to collectively identify and summarize the reported successes, challenges and lessons learned from the experience of engaging older adults with multimorbidity as research partners.

**Results:**

ACHRU researchers reported engaging older adult partners across many phases of the research process. Five challenges and lessons learned were identified: 1) actively finding patient partners who reflect the diversity of older adults with multimorbidity, 2) developing strong working relationships with patient partners, 3) providing education and support for both patient partners and researchers, 4) using flexible approaches for engaging patients, and 5) securing adequate resources to enable meaningful engagement.

**Conclusion:**

The lessons learned through this work may provide guidance to researchers on how to facilitate meaningful engagement of this vulnerable and understudied subgroup in the patient engagement literature.

## Background

Over the past two decades, the commitment to patient-oriented research and engaging patients as partners in research has steadily gained momentum across the healthcare system.^1,2^ Internationally and nationally, efforts to increase the inclusion of patients as partners in healthcare research have been driven by research funders,^2–4^ governments and patients themselves based on two main arguments. First, the moral argument is that research conducted on people without their input is unethical, particularly in marginalized communities.^4,5^ The second argument for patient engagement is the belief that patients’ lived experiences of health care offers unique and valuable perspectives that can increase the relevance, impact, and quality of research.^3,4,6^ Better understanding and incorporation of patient perspectives through early and continuous partnerships can result in patient-defined priorities, which are shown to lead to improved health outcomes, and a sustainable, accessible, and equitable healthcare system.^3,7,8^ The engagement of patients as partners in the design, implementation and evaluation of health research is now an expectation of several major international and national funding programs.^3,9^

Consistent with definitions from the Patient-Centred Outcomes Research Institute (PCORI) in the United States and the Canadian Institutes of Health Research (CIHR) Strategy for Patient-Oriented Research (SPOR) in Canada, the term “patient partners” within this article is intended to include patients, their caregivers, and members of the public who are directly involved as members of the research team, as opposed to being consenting research participants.^3^ The term “patient engagement in research” within this article is defined as “the active, meaningful, and collaborative interaction between patients and researchers across all stages of the research process, where research decision-making is guided by patients’ contributions as partners, recognizing their specific experiences, values, and expertise.”^1^

Engagement is especially important for hard-to-reach populations who are high users of the health care system, for whom data are often limited or missing because they are often excluded from research.^10^ Older adults (>65 years) with multimorbidity are one such population.^5,11^ Older adults with multimorbidity, defined as the co-existence of two or more chronic conditions in the same person,^12^ represent the largest growing segment of the population and the greatest users of the healthcare system. Worldwide, more than half of older adults have multimorbidity,^13^ with a mean of five chronic conditions per person.^14^ High prevalence rates of multimorbidity have been reported in older adults in Canada (43%),^15^ the US (63%),^16^ and the UK (67%),^17^ with a significant increase over the last three decades.^18,19^

Although multimorbidity is becoming increasingly common, health care is still organized to manage single diseases. This disadvantages patients, since treating each disease in isolation often leads to excessive treatment burden, polypharmacy, and fragmentation of care.^20^ Despite the existence of guidelines on multimorbidity care,^21^ there is limited evidence on how best to provide integrated community-based health and social services to older adults with multimorbidity.^20,22^ What works best when and for whom are largely missing in the multimorbidity literature.^23^ We need to enhance our understanding of multimorbidity using a broad range of different approaches. This will require not only investment in research, but also collaboration with patients and caregivers as partners to determine what outcomes matter most in the context of multimorbidity.^23^

Despite longstanding calls for greater engagement of older adults with multimorbidity as research partners, emerging evidence to suggest that this population can be successfully engaged,^24^ and their documented interest in participating in research, their input is under-represented.^5,23^ Older adults with multimorbidity are a diverse group of patients, ranging from relatively healthy, independent living individuals to very frail individuals with poor physical functioning and cognitive problems, which often can make engagement in research challenging.^5,24^ The evidence base to support engagement of populations similar to older adults with multimorbidity in healthcare research is sparce.^24^ Published reviews on patient engagement in research in the general population conclude that while engagement is feasible, more research is needed to understand exactly how and when to optimally engage patients.^8,25,26^ Funders, such as INVOLVE, PCORI and SPOR have highlighted the need to more clearly develop strategies to promote meaningful engagement and develop methods for evaluating the implementation and impact of patient engagement on research outcomes.^4,7,8,27^ This “knowledge to action” gap between the “what” and “how” of patient engagement may potentially limit its implementation, ongoing development, and uptake by researchers.^27^ The vulnerability of older adults with multimorbidity provides additional complexity to the engagement process, and practical issues that often deter researchers from involving similar populations in the research.^24^

Previous reviews have specifically explored guiding principles, and barriers and enablers to engaging a general population of older adults,^11^ and older adults with frailty ^5^ and dementia,^28^ in healthcare research. While this work offers an excellent starting point, engaging older adults living with multimorbidity and their family caregivers have specific challenges that require special consideration as we engage with this particularly vulnerable group. This includes challenges related to engaging older adults with multimorbidity deemed to be at-risk based on their health determinants, including low education, low income, and individuals living in rural settings.^5^ Further research is urgently needed to identify effective strategies to engage and partner with this vulnerable and understudied subgroup in the patient engagement literature both realistically and effectively.

### The Aging, Community and Health Research Unit

In 2013, our group developed the Aging, Community and Health Research Unit (ACHRU), a patient-oriented, pan-Canadian research program (https://achru.mcmaster.ca/). Initial funding for the establishment of ACHRU was provided by the CIHR Signature Initiative in Community-Based Primary Health Care and the Ontario Ministry of Health and Long-Term Care, Health System Research Fund, Canada. Since 2013, more than $11 million in additional leveraged funding has been raised from other national, provincial, and local funding agencies. The goal of ACHRU is to design, implement, evaluate, and scale-up innovative community-based interventions to improve quadruple aim outcomes (health outcomes, patient and provider experience, and costs) for older adults with multimorbidity and their family caregivers. The research unit consists of over 65 interprofessional researchers from 6 provinces, and over 200 stakeholders (health care providers, policy makers, and patient/caregiver research partners) from over 40 communities across Canada.^22^ As of 2020, the ACHRU’s research program included a total of 17 projects (13 completed, and 4 active projects). We use an integrated knowledge translation approach, based on the Knowledge-to-Action Framework,^29^ which involves engaging multiple stakeholders (patients, providers, policy makers) in all stages of the research program.^22^ Since the inception of ACHRU in 2013, over 40 older adults from across Canada have been involved in AHCRU projects as patient research partners (https://achru.mcmaster.ca/).

## Objectives

An important contribution of the ACHRU is the engagement of older adults with multimorbidity and their caregivers as research partners. We have gained many valuable insights regarding the engagement of this population as research partners. As such, the aim of this paper is to describe the successes, challenges, and lessons learned from this patient-oriented program of research on how to effectively engage older adults with multimorbidity as patient research partners.

## Methods

### Patient engagement approach in ACHRU

Our approach to patient engagement is aligned with the Knowledge-to-Action Framework.^29^ We strategically engaged patient partners throughout ACHRU’s governance structure (as members of advisory, stakeholder and steering committees and local community advisory boards for individual studies), as members of the research team as co-investigators, and as participants at workshops and training sessions. The patient engagement approach in ACHRU was guided by the CIHR SPOR Patient Engagement Framework ^3^ that embraces the principles of inclusiveness, support, mutual respect, and co-build.^30^

We used multiple strategies to find patient partners with personal knowledge and life experience with multimorbidity or the specific health condition being studied. This included: 1) identifying patient partners through our existing networks, 2) identifying patient partners who previously participated in our studies as research participants, 3) partnering with organizations who serve older adults with multimorbidity who assisted us in finding patient partners, and 4) leveraging relationships with other health care professionals within the health care system. The selection of patient partners took into account their motivation, willingness and ability to contribute to the research, and ability to move beyond simply sharing their personal experience to applying their experience in a contributory way.^25^ Based on feedback from our patient partners, we developed a brief, lay-language role description to support finding and establishing patient partners. This included providing information on the research, the role and expectations of patient partners, and strategies that were available to address potential barriers to partnering, (e.g., lack of transportation, respite care, and access to technology).

We identified a lead for patient engagement for each study who helped to build personal connections between patient partners and the research team and provided partners with ongoing support and mentorship. The leads were supported by the ACHRU patient engagement coordinator who provided oversight for all patient engagement activities from study-specific to program-wide activities. We took the time to gain an understanding of the preferences and expressed needs of our patient partners to optimize their participation in all stages of the research and share their expertise. This included identifying their choice of communication (in-person vs. telephone meetings, e-mail vs. postal mail). We assessed the alignment between individual and project goals and communicated with them regularly to identify potential roles in the research, expectations, preferred level of engagement, time commitment, and potential barriers to their participation in the research. For example, the location for engagement activities needed to be accessible to those with mobility challenges or for those facing transportation barriers.^24^ We used web-based communication platforms, e.g. Zoom, WebEx, as an alternative strategy when in-person meetings were not possible. We used a variety of strategies (e.g., small- and large-group discussions and activities) to optimize the participation of patient partners in all stages of the research and share their insights and expertise. We put multiple structures in place to ensure regular communication with patient partners about new developments, planned projects, research progress, research results, and new opportunities for participation.

Most of our patient partners did not have experience as a research partner or lacked knowledge of the research process. Initially, most of the researchers on our team also lacked experience working with patient research partners. To address this gap, arrangements were made in the early years of our research program, for all our researchers, trainees, decision-makers and patient partners to attend a 1-day workshop on patient engagement that was led by a researcher from INVOLVE in the UK. The workshop covered topics such as the value and importance of patient engagement, communication skills, and strategies to enhance researchers’ skills in how to meaningful and purposefully engage patient partners. This workshop laid the foundation for the development of a formal patient engagement training program that was offered to all our patient partners. The training was guided by the CIHR ethics guidance for developing partnerships with patients and researchers,^31^ and included information about the research project, some basic background on health research methods to allow them to participate and understand the discussions, and an overview of the role of patient research partners, and the breadth of ways in which patient partners can become involved in research.

As our funding and experience with patient engagement accumulated, we developed a customized patient engagement training program. We co-developed resources together with our patient partners to support our training strategy. These included lay-language descriptions of the research process and guidelines for patient partnering (https://achru.mcmaster.ca/). Further training and support related to specific tasks and roles were provided by the researchers. Ongoing training sought to leverage the strengths and abilities of the patient partners not with the intention of training patient partners as researchers, but to support their unique insights related to the research project. The patient partners received payment for their involvement. We utilized several resources to guide remuneration of our patient partners, including guidelines from the Ontario SPOR Support Unit,^32^ CIHR^33^ and Diabetes Action Canada.^34^ Patient partners were given the choice of how they wanted to be compensated (e.g., salary, honorariums, gift cards).

### Developing the lessons learned

We developed this list of lessons learned through iterative consensus-building and ongoing dialogue with ACHRU researchers and patient partners during the 7-years of the research program. Data used to identify this preliminary list included project documentation (such jas protocols), minutes of meetings and a workshop on patient engagement, field notes and observations made by researchers and patient partners after meetings, documented feedback after meetings, and resources developed to support patient engagement. An initial list of challenges and lessons learned was generated by the lead author (MMR). This was then iteratively discussed with all authors (including patient partner, GHT) until consensus was achieved regarding common challenges to patient engagement, and the lessons learned from the experience of engaging older adults with multimorbidity as research partners. The similarity of our lessons to the existing literature on patient engagement in both the general population of older adults, and in older adults with multimorbidity more specifically, will be discussed.

## Results

### Characteristics of patient partners in ACHRU

Since the inception of ACHRU in 2013, over 40 older adults from across Canada have been involved in the 17 AHCRU projects as patient research partners. Seventeen (42.5%) patient partners were males, 12 (30%) were family caregivers, and about one-third (32.5%) were members of the public.

### Patient partner research activities

Most projects (14/17; 82%) involved at least one patient partner, three (19%) involved two or more patients, and three (19%) involved six or more patients. Almost one-half (44%) involved patient partners as members of the research team as co-investigators. Most relationships between patient partners and researchers existed for at least 1 year. However, some researcher-patient partner relationships were more longstanding (>3 years). Patient engagement took many forms (different types of contributions on an ad hoc or ongoing basis), involved different patients in different roles, and occurred at different levels (e.g., program or project-level) and over different time periods (e.g., weeks, months, years). Seventeen (17.5%) patient partners were involved in research activities at the individual project-level as well as the broader ACHRU Program Steering Committee level. Within these roles, patient partners have been involved in a variety of activities in different stages of the research cycle, from identifying research priorities and questions, assisting with grant applications, co-designing interventions, informing real-time adaptations to the interventions, informing recruitment and consent processes, identifying patient- and caregiver-relevant outcomes and experience measures, interpreting findings, and contributing to the research program’s knowledge translation plan and products (e.g., presentations, videos, journal manuscripts). Further details on patient engagement within ACHRU^22^ and 1 of the 17 projects has been published elsewhere.^22,35,36^

The International Association of Public Participation (IAP2) spectrum of public participation denotes five levels of engagement ranging from “inform,” which involves letting patients know about the research to “consult,” where the goal is to obtain feedback from patients, to “involve” and “collaborate,” where researchers work directly with patients to share decision-making power, and finally to “empower,” where all decision-making is made by patients who actively control, direct and manage the research process.^37^ In the early stages of our research program, we primarily “informed” patients about the research or “consulted” with patients on their experiences to inform the research agenda. This level of involvement predominantly involves a flow of information in one direction, from patient partner to researcher.^38^ As our experience with patient engagement accumulated, we progressed along the IAP2 spectrum to more fully “involving” and “collaborating” with our patient partners. These levels of involvement involve two-way knowledge exchange between patient partners and researchers.^38^ No studies involved engagement of patient partners at the level of empowerment. The highest level of engagement was achieved in 4 of 17 studies (24%) where we collaborated with our patient partners across all stages of the research process from research priority setting, assistance with grant applications, input into study design, co-design of project materials, recruitment and consent strategies, data analysis, dissemination activities, and decision-making at research steering/advisory committees.

## Lessons learned

### 1) Actively finding patient partners who reflect the diversity of older adults with multimorbidity

An ongoing challenge we faced was finding patient partners who reflected the diversity of older adults with multimorbidity. Little is known about the resources and strategies needed to find patient partners, as well as the barriers and enablers to establishing patient partners from this vulnerable patient population.^39^ We learned the importance of using multiple strategies to recruit this population. We learned the value of applying equity, diversity, and inclusion principles to ensure representation of patient partners from under-represented, hard-to-reach subgroups of the older adult population who face unique challenges based on their health determinants (e.g., low income, lower level of education, poor health, or functional limitations) (https://cihr-irsc.gc.ca/e/51709.html).

The existing literature reports that a clear description of the role, responsibilities, commitment and benefits of participating in specific research projects is helpful for finding and establishing patient partners.^39^ A frequently reported barrier to engagement is the lack of public awareness about the need and (potential) impact of patient engagement in research, and what is expected of patients as co-researchers.^40^ We learned the importance of working with our patient partners to create clear expectations for patient roles and responsibilities and identifying and addressing potential barriers to partnering. We learned that the format of the information was vital, and factors such as literacy levels, language, education level, and age-associated decline need to be taken into consideration to improve its accessibility.^11^

### 2) Developing strong working relationships with patient partners

The establishment of genuine, reciprocal relationships between patient partners and researchers, underpinned by mutual trust, respect, clarity in roles to be undertaken, and valuing of different views and perspectives was fundamental to patient engagement in our research program. This is consistent with the literature that emphasizes the importance of building trusting relationships between patients and researchers, regardless of the length, type, or intensity of their engagement.^11,24,41,42^ Building these relationships took time, and opportunities for patient partners to contribute to the research were provided throughout the lifecycle of our projects.

The importance of regular contact and ongoing support and feedback has been identified as a key requirement for the establishment of effective partnerships.^24^ A key enabler of engagement was investing time to gain an understanding of the unique and complex challenges our patient partners faced, to ensure that their practical and emotional needs were addressed throughout the course of engagement, e.g. assisting with the logistics of attending meetings.^24^ Holding in-person full team meetings as early as possible during the research was also important to give patients and researchers the chance to get to know each other. We learned the importance of having a lead contact for each study who contacted patient partners on a regular basis and provided them with ongoing support, feedback, and briefing and debriefing before and after team meetings.

We learned the importance of creating a variety of opportunities for patient partners to share their insights and opinions and engage with other collaborators and researchers. For example, we used small- and large-group discussions and activities to create different opportunities for patient partners to share their insights and opinions. Other strategies that helped to optimize engagement included having patient partner input as a standing agenda item for all meetings, and allowing ample time for patient partners to ask questions during meetings to ensure that patient partners did not feel rushed or inhibited in any way. Holding face-to-face meetings, building-in time in the agenda for researchers and partners to socialize, and avoiding the use of jargon, acronyms and clinical or academic language were also important,^42,43^

A commonly cited barrier to meaningful patient engagement is that a patient’s role in research may be merely symbolic, often referred to as “tokenistic,” resulting in devaluing patients’ input.^8,25^ The development of genuine, trusting relationships between researchers and patient partners was key to combatting tokenism. For patient partners, the development of strong working relationships was underpinned by trust; trust was created when patient partners felt that their input was valued.^24^ We learned that this sense of value was enhanced when there was explicit appreciation by the research team of their contributions. To this end, we used diverse strategies to publicly in acknowledge the value and worth of our patient partners’ contributions, including: 1) skillful facilitators who ensured that patients’ contributions were recognized and valued during meetings, 2) public mention and acknowledgment of the value of the patient perspective in all of our research materials, 3) acknowledgment of our patient partners in our publications, and through co-authorship on articles, and co-presenting at academic presentations, 4) financial compensation for patient partner time, and 5) reporting back to patient partners about how their input was used in the research.

Aligned with these strategies, the literature suggests that aligning patient skills and interests with their roles are important factors for facilitating effective patient partnerships.^42,44^ We learned the importance of investing significant time to understand our patient partners’ stories, how and why they got involved in the research, what they hoped to bring to the project, what they hoped to get out of it, and what they hoped the project contributed. We assessed the alignment between individual and project goals and engaged in initial and ongoing communication with patient partners to clarify role, expectations, preferred level of engagement (from passive to active roles), and time commitment. This is consistent with other studies that stress the importance of matching patient experience, skills and background to the specific needs of the research to ensure that patient contributions are valued.^43^

Lack of role clarity and expectations related to the contribution of patient partners in the research are commonly cited barriers to meaningful engagement by both patients and researchers.^24^ We learned the importance of working with our patient partners to create clear expectations for patient roles and responsibilities and were careful not to make assumptions about what roles patient partners can fulfill.^35^ We continuously explored new opportunities for patient engagement within specific research projects and the research program.

A major challenge to engagement in research is the power imbalance which may exist or be perceived to exist between researchers and patient partners.^5^ We learned that addressing this potential power imbalance was fundamental to creating strong working relationships with our patient partners.^31^ Perceived power imbalances can be related to multiple factors, including differences in community or social status or expertise, differences in expertise or experience with research, economic hardship or poor health that prevents patients from fully engaging as partners in the research or differences in culture and expectations regarding appropriate ways of interacting.^3^ We used a variety of strategies to equalize this power imbalance that have been identified by many groups, including: 1) providing financial compensation for patient partners’ time and expertise, 2) ensuring equal participation of researchers and patient partners during meetings, and 3) sharing information and decision-making power with patient partners.

### 3) Providing education and support for both patient partners and researchers

One of the most common barriers to implementing effective patient engagement is the lack of training for both patient partners and researchers on engagement.^42,45^ Patients rarely have experience in health services research and can find the expectations of the role daunting or can be confused about their role as co-researchers versus participants being researched. This can lead to misunderstandings about why they were involved in the research or disappointment that they were not given support in how to manage their healthcare needs.^26^ The need for adequate training of both researchers and patients on engagement has been identified by many groups as an essential element for success.^3,45,46^ Providing basic training for patients in research methods has been cited by both patient partners and researchers as a key facilitator for building patients’ confidence in contributing to research and partnership.^24^ We learned the value of training both researchers and patient partners in patient engagement practices. The initial and ongoing training that we provided to these groups laid the foundation for establishing a culture of mutual trust and respect, understanding of perspectives, familiarity with terminology, and equitable participation.^31^

As the practice of patient engagement was a learning experience for all involved, we learned the importance of providing ongoing mentoring and coaching opportunities to both patient partners and researchers. All patient partners and researchers expressed growing confidence in their roles over time. Evidence for this was seen in how patient partners demonstrably shaped different aspects of the research process. Evidence for this was also seen in how researchers purposefully constructed meeting agendas to ensure time for patient partners to participate, and provided patient partners with opportunities to co-lead committees, and co-present at meetings and conferences.

### 4) Using flexible approaches for engaging patients

Many of our patient partners who were interested in partnering in research faced logistical barriers to their involvement, such as the need for respite care, access to technology, transportation, geographical barriers, poor health, or mobility limitations. A key learning from this experience was that engagement opportunities needed to be flexible with respect to the location, timing, and role in the research to accommodate individual needs and preferences.^35^ We learned the importance of gaining an understanding of the characteristics, demographics, preferences, and needs of our patient partners and identify any potential barriers to participation in the research.

We also learned the importance of being mindful of the potential burden associated with involvement in the research, and to be flexible with the time and task commitment to accommodate any changes in patients’ physical, cognitive, or emotional health or other life circumstances which may alter their ability and interest in engaging in the research.^5,11,24^ This was achieved through regular communication between researchers and patient partners to monitor any changes in their needs and identify and address any barriers to their ability to participate in the research, (e.g., mobility impairments or other physical challenges, accessing transportation, lack of familiarity with technology or the need for respite care). Family caregivers also needed to alter their level of engagement as a result of the changing health needs of the patient or themselves.^5^ We took as a guiding principle the belief that patient engagement is not a one-size-fits-all approach; customization is required. This often involved engaging different patient partners at different points for different tasks during the projects. The key learning from this experience was that being flexible and tailoring the type and intensity of engagement to individual needs and preferences ultimately helped to strengthen the relationship between the researchers and patient partners.

### 5) Securing adequate resources (time and funding) to enable meaningful engagement

Ensuring an equitable and sustainable compensation mechanism to recognize patient partners for their time and travel was an ongoing challenge in our research program. Resource constraints are known to be another barrier to implementing effective patient engagement.^4,24,42^ Inherently, compensation, or the lack thereof, can contribute to a lack of trust and power imbalance between researchers and patient partners.^42,45^ From an equity perspective, compensation can also enable the participation of marginalized older adults with limited financial resources. Comprehensive compensation policies can overcome these barriers.^47^ Many groups have stressed the importance of compensating patient partners for their time and the skills and experience they bring forward. In the early years of the research program, resource constraints, including funding, human resource capacity for support, and time constraints were major barriers to engaging patient partners.^24,45^

The introduction of SPOR-funded projects to our research program that required and funded patient engagement was a key enabler to the implementation of our patient engagement strategy. This change in granting guidelines and the funding that resulted, helped to offset the time and costs associated with engaging patient partners, including the costs to support the ACHRU patient engagement coordinator and the patient engagement lead on each study. The funding allowed us to further expand our patient engagement strategy to include more patient partners and to move further along the engagement spectrum.^37^ The learning from this experience is that appropriate support (financial, human) must be made available to teams dedicated to engaging older adults with multimorbidity as research partners. Partnering with older adults with multimorbidity will invariably involve additional investments of time, money, and human resources to compensate for the accompanying burden involved in this endeavor. The patient engagement plan for our individual studies now includes more realistic allowances for patient engagement costs and time in the budgeting and planning of the research (e.g., both remuneration costs and dedicated personnel time to support meaningful engagement).

Despite the introduction of funding for engagement, we encountered several challenges with respect to determining how and when our patient partners were to be compensated for their participation. Generally, the guidelines for compensating patient partners suggests that remuneration be aligned with the level of involvement of patient partners within the study. However, complexities around payment existed for patient partners who were low income and depended on social supports with income qualifications. If these patient partners were paid in this way, the extra income might disqualify them for this benefit. Working in partnership with our patient partners, we moved to a different compensation model that was still fair and equitable but did not need to be the same for everyone. The learning from this experience is that we now have open conversations with our patient partners and give them the choice on how they wanted to be compensated (e.g., salary, honorariums, gift cards).

A further challenge related to remuneration of our patient partners was creating a compensation model that reimbursed patients as a research team member and not research participants. In the absence of University-level guidelines for compensating patient partners, we needed to consent our patient partners as research participants to reimburse them for their time on the research. These experiences highlight the need for a standardized national process for compensating patients as research partners that supports shifting the lens from “paternalistic ideals of consenting patient partners to studies ‘owned’ by researchers to a mutually beneficial partnership between those with lived experience and those researching that experience”^42^ (p. 536).

## Strengths and limitations

While some of the challenges and lessons learned reported here are similar to those reported in other literature on patient engagement, this paper is unique in that it: 1) highlights the unique challenges and strategies of partnering with older adults with multimorbidity, an understudied subgroup in the patient engagement literature, and 2) captures this information across a group of studies within a multi-year program of research. The lessons reported here are also unique in that they provide guidance for researchers to engage patient partners throughout the research process. Most efforts to engage patient partners continue to be limited to the early stages of the research, during study design and recruitment, rather than in the later stages of the research process.^48^

Conversely, there are limitations to the paper that warrant acknowledgment. The research projects that included patient engagement activities were carried out in six provinces across Canada, and thus, the findings may not be generalizable to other provinces or settings outside Canada. We secured funding to offset some of the costs of engaging older adults as research partners, including patient compensation, development of recruitment and orientation materials, and securing time and resources for research staff to support patient engagement activities. These resources would need to be found in future research to act on the lessons learned through this research program. The successes, challenges and lessons learned reported in this paper are based on anecdotal feedback from our researchers and patient partners. A more formal evaluation of the implementation and impact of ACHRU’s patient engagement strategy is currently underway.

## Conclusions

Engaging older adults with multimorbidity as research partners has provided our research team with unique insight into what it is like to live with multimorbidity to develop research that more accurately addresses their needs. While engaging older adults with multimorbidity as research partners presents challenges,^24^ our experience suggests that engaging this population is feasible, and the challenges can be overcome. Very few studies have engaged older adults with multimorbidity, who have unique needs due to their complex health and social conditions. Here, we have described different experiences of integrating this population as research partners drawing from real-world examples. These diverse experiences highlight the successes and challenges of partnering with patient partners across the research process, from conceptualizing, designing and conducting research, to disseminating findings.

The lessons learned from this patient-oriented research program on how to engage older adults with multimorbidity and their caregivers as research partners (e.g., role clarity, trust and willingness to collaborate, capacity building for both patients and researchers, funding, ongoing support) are similar to those reported by others.^5,11,24,28,35,42,45^ However, our experience contributes additional learning that addresses the specific challenges related to partnering with this particularly vulnerable group. These include, but are not limited to: 1) building strong personal connections within the team, underpinned by mutual respect, trust, co-learning and valuing of patient partner contributions; 2) having a lead contact for each study who built person connections with patient partners, communicated regularly with patient partners, and provided patient partners with ongoing support and mentorship; 3) ensuring that the practical and emotional needs of patient partners are addressed; 4) being mindful of the potential burden of research-related activities and using flexible approaches to accommodate individual needs and preferences (e.g., location, timing and role in the research) and changes in health status or caregiving demands; 5) addressing training needs of all team members on patient engagement; 6) ensuring clarity in patient partners’ roles and their expected contribution; and 7) identifying and securing adequate resources (time and funding) to facilitate and sustain patient partnering activities. These strategies may also help to explain how movement of our engagement practices along the IAP2 engagement spectrum, from merely “informing” patients about the research to fully “involving” or “collaborating” with patient partners—the former, a subject of criticism, and the latter, promoted as an ideal (39) was achieved.

## Future directions

Over the past 7 years, as we continue to increase and diversify our patient partner group in ACHRU, we not only recognize the valuable role patients play in our research, but also have made a deep commitment to advance the science of patient engagement. In their recent scoping review of patient engagement in Canada, Manafo and colleagues reported that the existing evidence base to support patient engagement is limited to “lessons learned,” recommendations and checklists.^27^ While our experiences can serve as a guide for optimizing patient research partnerships, further research is needed to identify the approaches that best support effective patient research partnerships, the types of activities that are meaningful to patient partners, as well as approaches to involve older adults in co-designing strategies for meaningful engagement.^49^ We also need research that examines the impact of different patient engagement strategies on patient-relevant outcomes, such as quality of life, self-management of their own conditions, and increased knowledge of the health care system. A more formal evaluation of the implementation and impact of ACHRU’s patient engagement strategy is underway. Such research could advance patient engagement in older adults with multimorbidity, ultimately providing a much-needed evidence-base for the development of best practices for patient engagement and the provision of high-quality care.
